# 1-Year Outcomes of a Multicenter Randomized Controlled Trial of the Ankura II Thoracic Endoprosthesis for the Endovascular Treatment of Stanford Type B Aortic Dissections

**DOI:** 10.3389/fcvm.2022.805585

**Published:** 2022-03-15

**Authors:** Chang Shu, Hao He, Weiguo Fu, Wei Guo, Ming Li, Erping Xi, Shuguang Guo, Xueming Chen, Zhanxiang Xiao, Shiqiang Yu, Jianhua Huang, Xiangchen Dai, Zhiwei Wang, Wei Li, Qingshan Zheng, Quanming Li, Lunchang Wang, Xin Li, Junwei Wang, Feng Gu

**Affiliations:** ^1^Department of Vascular Surgery, Second Xiangya Hospital, Central South University, Changsha, China; ^2^Department of Vascular Surgery, Fuwai Hospital, Chinese Academy of Medical Science & Peking Union Medical College, Beijing, China; ^3^Department of Vascular Surgery, Institute of Vascular Surgery, Zhongshan Hospital, Fudan University, Shanghai, China; ^4^Department of Vascular and Endovascular Surgery, Chinese PLA General Hospital, Beijing, China; ^5^Department of Vascular Surgery, The First Affiliated Hospital of Medical School of Zhejiang University, Hangzhou, China; ^6^Department of Vascular Surgery, Wuhan General Hospital of Guangzhou Military Region, Wuhan, China; ^7^Department of Vascular Surgery, Kunming General Hospital of Chengdu Military Region, Kunming, China; ^8^Department of Vascular Surgery, Beijing Friendship Hospital, Capital Medical University, Beijing, China; ^9^Department of Vascular Surgery, Hainan Provincial People's Hospital, Haikou, China; ^10^Department of Vascular Surgery, The First Affiliated Hospital of the Fourth Military Medical University, Xi'an, China; ^11^Department of Vascular Surgery, Xiangya Hospital, Central South University, Changsha, China; ^12^Department of Vascular Surgery, General Hospital of Tianjin Medical University, Tianjin, China; ^13^Department of Vascular Surgery, Wuhan University People's Hospital, Wuhan, China; ^14^Medical Research and Statistics Center, Fuwai Hospital, Chinese Academy of Medical Sciences, Beijing, China; ^15^Drug Clinical Research Center, Shanghai University of Traditional Chinese Medicine, Shanghai, China

**Keywords:** Ankura II, RCT, thoracic endovascular aortic repair, type B aortic dissection, stent – evolution

## Abstract

**Background:**

The Ankura II Thoracic Stent Graft System (Lifetech, Shenzhen, China) is an evolution of the Ankura stent graft. This study reports one-year outcomes of the Ankura II Thoracic Stent Graft System for endovascular treatment of Stanford type B aortic dissections.

**Methods:**

The Ankura II Thoracic Aortic Endovascular Trial was a randomized, single-blinded, clinical trial conducted at 12 Chinese institutes. The enrolled patients diagnosed with Stanford type B aortic dissections (TBADs) were randomly assigned to the Ankura group or Ankura II group. Standard follow-up examinations were performed at 1, 6, and 12 months. Safety and efficacy data were analyzed.

**Results:**

132 patients with TBADs were enrolled. The outcomes for the primary safety end points revealed that the Ankura II stent graft was statistically non-inferior compared to the Ankura stent graft. The 1-month device-related major adverse events (1.6 vs. 0%; *p* = 0.48), 1-month all-cause mortality (1.7 vs. 4.5%; *p* = 0.621), 12-month survival rate (95.2 ± 2.7% vs. 94.1 ± 2.9%; *p* = 0.769), and major adverse event (MAE) rate (5.1 vs. 4.7% at 1 month; *p* = 0.73 and 5.8 vs. 8.9% at 12 months; *p* = 0.718) of Ankura II group are all comparable to Ankura group. The two groups showed similar primary effectiveness and true lumen expansion effect, and false lumen remodeling was improved in Ankura II group (−100.0 vs. −48.5%; *p* = 0.08).

**Conclusions:**

The one-year outcomes from this prospective, randomized, multicenter study demonstrate that Ankura II stent graft shows comparable results to Ankura for treating TBADs, resulting in low mortality rates, MAEs and reintervention rates.

**Clinical Trial Registration:**

ChiCTR-TRC-12002844.

## Introduction

The thoracic endovascular aortic repair (TEVAR) has been well-studied and demonstrated it can improve the early treatment outcomes of thoracic lesions compared to conventional best medical therapy (BMT) and open surgery (1, 2). The several pioneering thoracic stent graft clinical trials established TEVAR combined with BMT as a safe and effective strategy for treating Stanford type B aortic dissection (TBADs), especially for high risk and complicated patients (3–5). Recently, TEVAR was extended to manage stable uncomplicated type B dissection due to the potential role of remodeling dissected aorta and preventing late expansion and malperfusion (6, 7). The booming of TEVAR really changes the treatment therapy of thoracic lesions.

However, the current commercialized stent graft design still faces some anatomic challenge, since the diameter of thoracic aorta is decreasing from proximal end to distal end (8). The size mismatch between the distal end of the stent graft and the remarkably small diameter of a compressed distal true lumen (TL) may contribute to the distal endoleak or stent graft-induced new entry (SINE) (9, 10). Moreover, the relative short stent can cause incomplete false lumen (FL) thrombosis of the aorta, thus affecting vessel remodeling (11). Recent studies have shown that the degree of FL thrombosis and aortic remodeling are associated with better long-term outcomes of TEVAR (12, 13).

Given the challenge as we mentioned above, the Ankura II Thoracic Stent Graft System (Lifetech, Shenzhen, China), an evolution of the Ankura stent graft, is designed. Compared to first generation product, The Ankura II stent graft is available in straight or tapered configurations. And the new shape provides flexibility and conformability in the stent graft, exerting radial force to enhance seal and fixation. The old system has already showed early evidence of a safe, effective, and durable endoprosthesis for the treatment of descending aortic aneurysms (14). Yet no study has been done for the Ankura II system. Since the new system has many theoretical advantages and available to more patients, we conduct this non-inferiority study. Our goal is to present characteristics and early outcomes of this novel endovascular stent graft, Ankura II Thoracic Stent Graft System, for the treatment of TBADs. Our early data from multicenter in China reveals the safety and efficacy of the Ankura II stent graft in the treatment of TBADs and better aortic remodeling compared to Ankura stent graft.

## Materials and Methods

### Enrollment

The Ankura II Thoracic Aortic Endovascular Trial was a prospective, randomized, multicenter study designed to evaluate the safety and efficacy of the Ankura II Graft System for the treatment of thoracic lesion (Clinical trial registration number: ChiCTR-TRC-12002844). The study was approved by the Institutional Review Board of each participating institution. All participates were well-informed the potential benefit and risk and signed the consent. The trial enrolled 134 patients (two patients withdrew consent before surgery) with TBADs and 10 patients with aortic aneurysm from 12 institutions in China between November 2012 and December 2016. Since the sample size of aneurysmal patients was too small, only patients with TBADs were analyzed in this article to ensure homogeneous results. Data from aneurysmal patients can be provided to readers with reasonable require. The block randomization method was adopted, with a block size of four. The random envelopes were used. Patients were randomly assigned to the Ankura II group or Ankura group in a 1:1 ratio. The anatomic and medical inclusion and exclusion criteria were described in Table 1. All the anatomic enrollment criteria were assessed by independent clinical research associates and attending physicians together.

**Table 1:** Anatomic and medical inclusion and exclusion criteria.

***Inclusion criteria***
Age ≥18-years-old;
Diagnosed with Stanford type B aortic dissection, landing zone ≥20 mm distanceto the left subclavian artery (or the landing zone ≥20 mm distance to the left common cervical artery and the left vertebral artery is non-superior);
Patent femoral and iliac arteries or can tolerate a vascular conduit allowing endovascular access to dissection area via a delivery system;
• Life expectancy ≥1 year.
***Exclusion criteria***
Pregnant or breastfeeding women; Medical history of aortic surgery (open or endovascular); History of cardiac or cerebral infarction within 3 months; Diagnosed with connective tissue disease or active systemic infection; Severe coagulation dysfunction; Potential genetic deficiencies and congenital diseases; Trauma patients or patients using illicit drugs.


### Device Description

**Figure 1:**
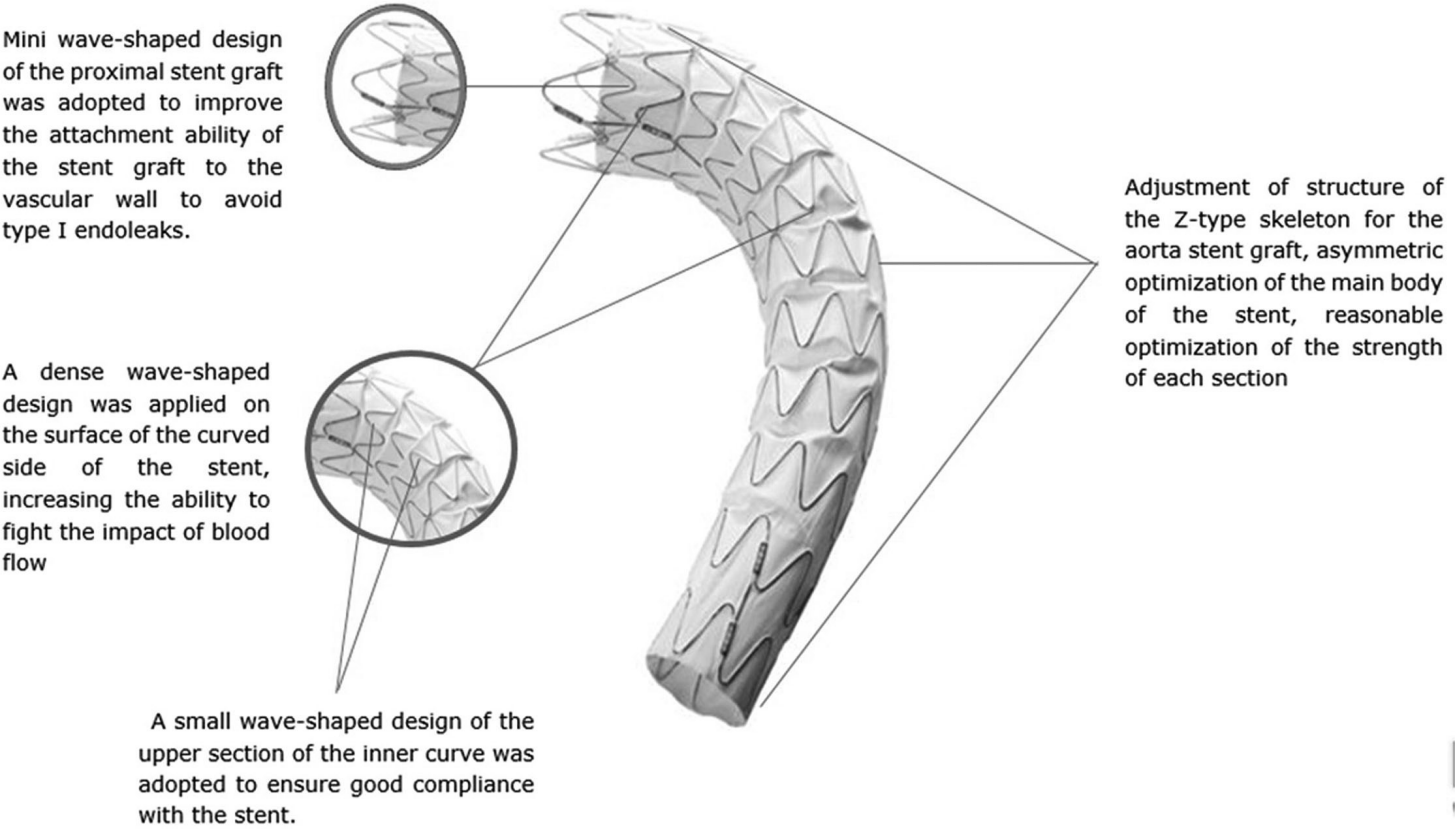
Overall observation of the Ankura II stent graft.

The Ankura II Thoracic Stent Graft System is a modular device comprising of a self-expanding metal stent dual-layer hot oxygen fusion polytetrafluoroethylene membrane with a bare proximal stent and covered distal stent. Based on the original Ankura stent graft, Ankura II includes some new improvements as shown in Figure 1. No suture was found on the main body to avoid pinhole leakage, thus providing better biocompatibility and durability. The sinusoidal shape and placement of the self-expanding nitinol springs also provide flexibility and conformability in the stent graft, exerting radial force to enhance seal and fixation. The Ankura II stent graft is available in straight or tapered configurations (6-mm, 8-mm or 10-mm taper) and is offered in diameters ranging from 24 to 42 mm and covered lengths ranging from 60 to 200 mm. The detailed deployment process is described in the Supplementary data. The consistency of treatment was tested after the deployment process.

### End Points and Definitions

The primary safety end point was device-related major adverse events (MAEs) rate at 1 month, which includes stent could not open successfully; stent thrombosis, migration, collapse, and fracture, accidental blockage of other branches of blood vessels, and stent-related endoleaks.

The secondary safety end points were all-cause mortality rates and MAEs rates at 1, 6, and 12 months after surgery. The definitions of MAEs were as follows: cardiovascular events [heart failure, myocardial infarction, cardiac tamponade, retrograde type A aortic dissection (RTAD), rupture of dissection and ischemia of left subclavian artery (LSA)], neurological events (stroke, paraplegia, and coma), acute kidney failure, respiratory failure needs non-invasive or invasive respiratory support, and death. The primary effectiveness end points were clinically successful treatments, which were defined as the absence of any type I or type III endoleaks. The FL thrombosis rates and diameter and area changes in the FL and TL were also compared at 1, 6, and 12 months after surgery.

Endoleaks were defined according to the well-established type I to IV nomenclature (15). Migration was defined as >10 mm proximal or distal movement of the stent graft relative to fixed anatomic landmarks.

### Data Management and Statistical Analysis

The standard follow-up protocol for the study included physical examination and computed tomography angiography (CTA) at 1, 6, and 12 months after surgery. We reported our data based on either intention-to-treat analysis (ITT) or per-protocol (PP) analysis. Due to the missing data, we cannot perform full set ITT analysis. For those who had missing category data, we consider the data from previous time follow-up as this time follow-up.

All data analysis and reports of aortic dissection followed the Society for Vascular Surgery and Society of Thoracic Surgeons' reporting standards (3). All imaging studies were performed at the participating sites and reviewed by the core laboratory, which was in second Xiangya hospital, Changsha, Hunan, China. To assess the change and remodeling of the aortic lumen during follow-up, three different representative aortic planes were selected. Plane 1 was the aorta plane with the maximal diameter. Plane 2 was the aorta plane where the TL was most constricted by the FL. Plane 3 was the aorta plane of the distal end of the stent.

All deaths and MAEs were reported by the participating site and adjudicated by the clinical events committee to determine whether they were secondary to failure of the device, related to the procedure, or both.

The normally distributed continuous variables were expressed as mean ± standard deviation and other continuous variables were presented as median (interquartile range). Categorical variables were expressed as proportions (percentage). The two-sided Student's *t*-test and Mann-Whitney *U* test are used to compare continuous variables. And Chi-square test or Fisher's exact probabilities were used to compare category variables. The cumulative survival estimates were generated by using the Kaplan-Meier non-parametric method and compared using the log-rank test. The primary safety end point, the device-related MAEs rates at 1 month, was submitted to a non-inferiority comparison with a null hypothesis that Rate_AnkuraII_-Rate_Ankura_ ≤ -.1. (The rate refers the risk of the MAEs)

The sample size calculation was based on power analysis. According to a previous report, the incidence of device-related MAEs was.05 in the control group at the 1-month follow-up (16–18). Based on statistical requirements (unilateral α =0.05, β = 0.20, non-inferiority margin δ = 0.1), 118 cases were required after calculation. Considering that the 1-month follow-up rate should not be lower than 90%, the total sample size required was 130 cases. In the trial, 132 dissecting patients were enrolled to ensure statistical power.

## Results

### Follow-Up Process and Results

**Figure 2:**
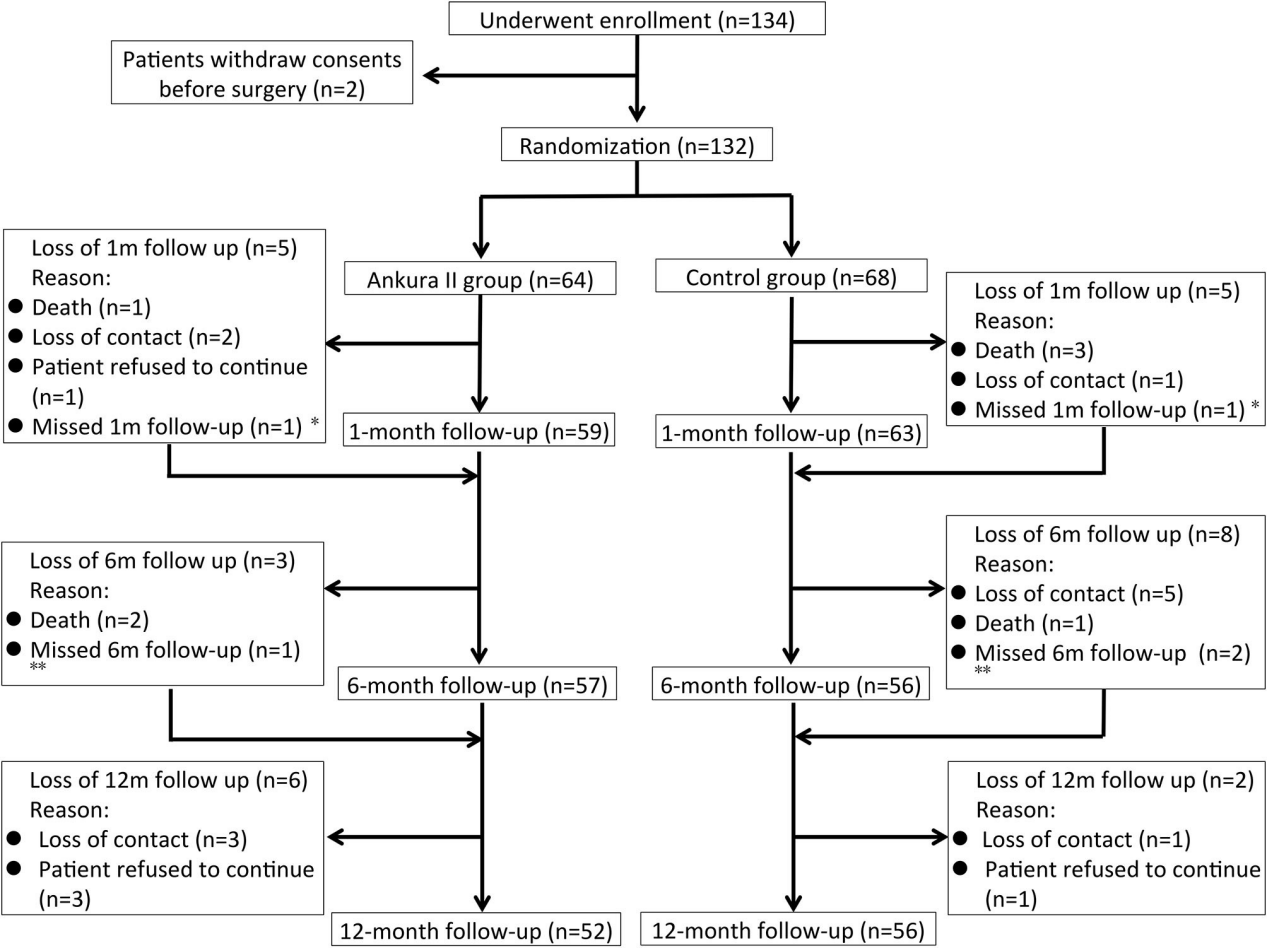
Illustration of the study cohort and the follow-up process. ^*^Ankura II group Patient No. 11 and Ankura group Patient No. 129 missed the 1-month follow-up but finished the 6- and 12-month follow-ups. ^**^Ankura II group Patient No. 117 and Ankura group Patient No. 50 and No. 120 missed the 6-month follow-up but finished the 12-month follow-up.

134 patients with TBADs were enrolled in the study, two of which withdrew consent before surgery. The study cohort and follow-up process are described in Figure 2. A total of 108 (81.8%) patients completed the 12-month follow-up, 7 patients (5.2%) died during the follow-up, and 17 (12.9%) patients were lost to follow-up. Of the patients who were lost to follow-up, 12 were unfortunately lost to contact, and five patients refused to continue in the study. Overall, 132 patients were included in ITT analysis and 108 patients were included in PP analysis.

### Demographics of the Enrolled Subjects

The demographics and risk factors showed no significant differences between the Ankura II and Ankura group (Table 2). Based on the definition of high-risk and complicated TBADs from Society for Vascular Surgery in 2020, we clarified the type of TBADs in both group by the data we had. The high risk of TABDs is defined as having an aorta diameter >40 mm or false lumen diameter >22 mm. The complicated TABDs is defined as existing ischemia of branch arteries. 66.2% of patients in Ankura group are high risk patients and 79.7% of patients in Ankura II group are high risk patients (*p* = 0.120). 11.8% of patients in Ankura group are complicated patients and 9.4% of patients in Ankura II group are complicated TABDs patients (*p* = 0.656). Besides, the rates of persistence of pain despite adequate blood pressure control and pain medications was 77.4% in Ankura group vs. 83.1% in Ankura II group (*p* = 0.499).

**Table 2:** Demographics of the enrolled subjects.

	**Ankura II %(n/N)**	**Ankura %(n/N)**	***P*-value**
Age (year)	56.4 ± 10.5	55.8 ± 11.7	0.85
Height (cm)	167.0 ± 8.2	166.9 ± 7.4	0.79
Weight (kg)	68.7 ± 12.3	66.8 ± 11.4	0.22
Male	81.3% (52/64)	83.8% (57/68)	0.70
Smoking	42.2% (27/64)	44.1% (30/68)	0.82
Cardiovascular history	65.6% (42/64)	64.7% (44/68)	0.91
Hypertension	64.1% (41/64)	61.8% (42/68)	0.78
Coronary artery disease	1.6% (1/64)	2.9% (2/68)	0.96
PCI	1.6% (1/64)	1.5% (1/68)	0.50
Medical treatment	65.6% (42/64)	64.7% (44/68)	0.91
Cerebral infarction History	3.1% (2/64)	1.5% (1/68)	0.96
Diabetes	6.3% (4/64)	4.4% (3/68)	0.93
COPD	1.6% (1/64)	0% (0/68)	0.48
Chronic renal failure	0% (0/64)	0% (0/68)	
Back pain	67.2% (43/64)	73.5% (50/68)	0.70
Chest pain	15.6% (10/64)	8.8% (6/68)	0.23
Abdominal pain	28.1% (18/64)	22.1% (15/68)	0.69
Asymptomatic	7.8% (5/64)	8.8% (6/68)	0.83
**High-rsik TBADs**			
Aorta diameter >40 or false lumen diameter >22 mm	79.7% (51/64)	66.2% (45/68)	0.08
Refractory pain	76.6% (49/64)	70.6% (48/68)	0.44
**Complicated TBADs**			
Ischemia of branch arteries	9.4% (6/64)	11.8% (8/68)	0.66
HR with medicine (bpm)	74.2 ± 10.5	74.5 ± 11.5	0.88
BP with medicine (mmHg)	127.0 ± 19.2/78.2 ± 12.0	126.4 ± 16.1/74.5 ± 9.4	0.85/0.050

*Bpm, beats per minute; BP, blood pressure, systolic/diastolic; COPD, Chronic obstructive pulmonary disease; HR, heart rate; PCI, Percutaneous coronary intervention; TBAD, Stanford type B aortic dissection*.

### Baseline Anatomical Features of Aortic Lesions

Comparable baseline anatomical features, including important anatomical factors such as ischemia of branch arteries, primary entry tear site, landing zone situation, and TL and FL diameter, were found between the Ankura II and Ankura group, Detailed information is listed in the Supplementary data and Supplementary Table 1.

### Surgical Procedures

The detailed results of the surgical procedures, including the deployment of stents, anesthesia methods, total surgery time, time for stent deployment, contrast agent volume and digital subtraction angiography (DSA) time, are described and compared (Supplementary data and Supplementary Table 2). No significant difference was found between the two groups.

### Device-Related Major Adverse Events, Mortality, and MAEs

Migration, collapse, fracture, or thrombosis of the stent was not found in either group during the 12-month follow-up, only one stent-related endoleaks (Type IV) occurred in the Ankura II group (Table 3). The incidence of 1-month device-related MAEs was 1.6% in the Ankura II group and 0% in the Ankura group. And the lower limit of the unilateral 95% CI between the Ankura II and Ankura group was -.055. According to the non-inferiority standard of -.1, the Ankura II stent system was non-inferior to the Ankura stent. No reintervention was required for device related MAEs in either group during the follow-up.

**Table 3:** Device-related major adverse events, mortality, and major adverse events.

	**1 month (ITT analysis)**			**12 months (PP analysis)**		
	**Ankura II (n/N)**	**Ankura (n/N)**	***P*-value**	**Ankura II (n/N)**	**Ankura (n/N)**	***P*-value**
Device-related MAEs	1.6% (1/64)	0% (0/68)	0.48	-	-	-
Stent migration	0% (0/64)	0% (0/68)	-	0% (0/52)	0% (0/56)	-
Stent collapse	0% (0/64)	0% (0/68)	-	0% (0/52)	0% (0/56)	-
Stent fracture	0% (0/64)	0% (0/68)	-	0% (0/52)	0% (0/56)	-
Stent thrombosis	0% (0/64)	0% (0/68)	-	0% (0/52)	0% (0/56)	-
Stent-related endoleaks (IV)	1.6% (1/64)	0% (0/68)	.48	1.9% (1/52)	0% (0/56)	0.48
Device-related re-intervention	0% (0/64)	0% (0/68)	-	0% (0/52)	0% (0/56)	-
1-month all-cause mortality	1.6% (1/64)	4.4% (3/68)	0.66	-	-	-
12-month survival	-	-	-	95.2 ± 2.7%	94.1 ± 2.9%	0.77^a^
Major adverse events	5.1% (3/64)	4.7% (3/68)	.73	5.8% (3/52)	8.9% (5/56)	0.72
**Cardiovascular events**						
Heart failure	1.6% (1/64)	0% (0/68)	0.48	1.9% (1/52)	0% (0/56)	0.48
Cardiac tamponade	0% (0/64)	1.5% (1/68)	>0.99	0% (0/52)	1.8% (1/56)	>0.99
RTAD	0% (0/64)	1.5% (1/68)	>0.99	0% (0/52)	1.8% (1/56)	>0.99
Rupture of dissection	0% (0/64)	1.5% (1/68)	>0.99	0% (0/52)	1.8% (1/56)	>0.99
Ischemia of LSA	0% (0/64)	1.5% (1/68)	>0.99	0% (0/52)	1.8% (1/56)	>0.99
**Neurological events**						
Stroke	1.6% (1/64)	0% (0/68)	0.48	1.9% (1/52)	3.6% (2/56)	>0.99
Paraplegia	1.6% (1/64)	0% (0/68)	0.48	1.9% (1/52)	0% (0/56)	0.48
Coma	1.6% (1/64)	0% (0/68)	0.48	1.9% (1/52)	0% (0/56)	0.48
Acute kidney injury	0% (0/64)	0% (0/68)	-	0% (0/52)	0% (0/56)	-
Respiratory failure	1.6% (1/64)	0% (0/68)	0.48	1.9% (1/52)	0% (0/56)	0.48
Endoleaks						
Endoleaks I	3.1% (2/64)	0% (0/68)	0.23	1.9% (1/52)	1.8% (1/56)	>0.99
Endoleaks II	1.6% (1/64)	0% (0/68)	0.48	0% (0/52)	0% (0/56)	-
Endoleaks III	0% (0/64)	0% (0/68)	-	0% (0/52)	0% (0/56)	-
Re-intervention rates	1.6% (1/64)	0% (0/68)	0.48	7.7% (4/52)	7.1% (4/56)	>0.99

*LSA, left subclavian artery; MAE, major adverse events; RTAD, retrograde type A aortic dissection*.

a *Log-rank test; ITT analysis, intention to treat analysis; PP analysis, per-protocol analysis*.

**Figure 3:**
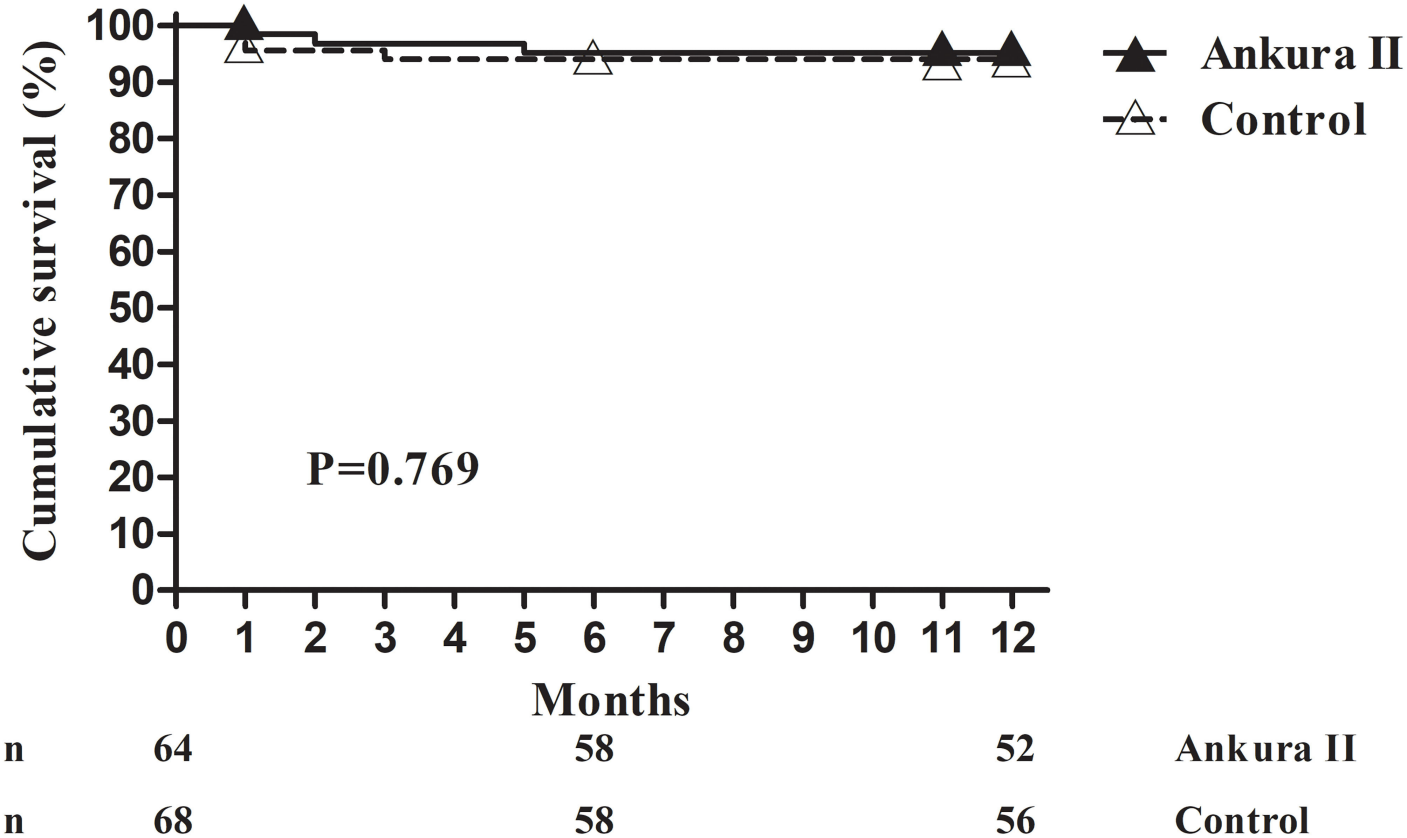
Kaplan-Meier survival analysis. The available number of patients between the Ankura II and Ankura groups was 64 and 68 at 0 months, 58 and 58 at 6 months, and 52 and 56 at 12 months, respectively.

The 1-month all-cause mortality rate for the Ankura II group was 1.6% (1/64), that for the Ankura group was 4.4% (3/68). Considering the loss of follow-up, the 12-month cumulative survival (Ankura II 95.2 ± 2.7% vs. Ankura 94.1 ± 2.9%; *p* = 0.769) was compared instead of the mortality rate. No significant difference was found between the two groups (Table 3). The Kaplan-Meier survival analysis with the exact time of death for Ankura II and Ankura patients is presented in Figure 3.

One patient in the Ankura II group exhibited paraplegia immediately after surgery and stroke after 5 days. Considering the poor prognosis, his family discontinued all treatment, and the patient unfortunately died. And there were three patients in the Ankura group who died within 1 month. One patient died 1 day after surgery with acute shock possibly due to rupture. Another patient died of acute pericardial tamponade, and RTAD 2 days after surgery. The third patient suddenly died at home 17 days after surgery with unknown cause.

Within 12-month follow-up, there were another two deaths in the Ankura II group. One patient died of multiple organ failure 50 days after surgery, and another died suddenly at home 168 days after surgery with unknown cause. In the Ankura group, one patient suddenly died at home of an unknown cause 65 days after surgery.

The distribution of MAEs at 1 month (ITT analysis, Ankura II: 4.7% (3/64): Ankura: 4.4% (3/68), *p* = 0.73) and 12 months (PP analysis, Ankura II: 5.8% (3/52): Ankura: 8.9% (5/56), *p* = 0.718) is shown in Table 3. In the Ankura II group, the cardiovascular events included a 1.6% (1/64) heart failure rate with respiratory failure within 1 month. In the Ankura group, the cardiovascular events included the rupture of dissection (1.5%), ischemia of the LSA (1.5%) and RTAD with cardiac tamponade (1.5%) occurring within 1 month. Neurologic events in the Ankura II group included paraplegia complicated with a later stroke (1.5%) and transient coma (1.5%) within 1 month. In the Ankura group, two stroke events occurred within the 12-month follow-up.

The endoleaks rate was low for both groups. At 1 month, the type I endoleaks rate was 3.1% (2/64) in the Ankura II group and 0% in the Ankura group (*p* = 0.232). At 12 months, the type I endoleaks rate was 1.9% (1/52) in the Ankura II group and 1.8% (1/56) in the Ankura group (*p* = 1.000), medical treatment was continued. Additionally, one type II endoleaks occurred in the Ankura II group within 1 month but disappeared during the 12-month follow-up. No type III endoleaks occurred.

The reintervention rates for 1 month (1.6% vs. 0%: *p*=.48) and 12 months (7.7 vs. 7.1%: *p* = 1.000) between the Ankura II and Ankura groups are also shown in Table 3 and no dissection or aneurysm vascular-related intervention occurred in either group. Detailed reintervention events included one lumbar disc herniation, one renal cancer, one multiple organ failure and one adrenal adenoma in the Ankura II group and two strokes, one kidney stone and one lung cancer in the Ankura group.

### Stent Effectiveness and Remodeling of TL and FL in TBADs Patients

The 12-month follow-up was completed for 52 patients in Ankura II and 56 patients in Ankura group (Table 4). To evaluate the effectiveness of stents and compare the ability of stents to expand the TL and facilitate FL thrombosis in AD patients (51/52 and 55/56 patients in the Ankura II and Ankura group, respectively), the FL thrombosis rate was compared. Three different aorta planes were chosen, and the diameter and area of the TL and FL were measured before surgery and at the follow-up in dissection patients.

**Table 4:** Effectiveness of Ankura II, remodeling of the aorta and percentage change in the TL and FL diameter and area in TBADs (PP analysis).

	**Ankura II (%)**	**Ankura (%)**	***P*-value**
Clinical successful treatment	51/52 (98.1%)	54/56 (96.4%)	>0.99
**FL thrombosis rate**			
Partially, 1 month	27 (45.8%)	27 (43.5%)	0.81
Completely, 1 month	32 (54.2%)	35 (56.5%)	
Partially, 12 months	20 (39.2%)	15 (26.8%)	0.15
Completely, 12 months	31 (60.8%)	41 (73.2%)	
**12-month follow-up**			
**Plane 1**			
Diameter of TL	36.4 (8.1–102.5)	48.2 (23.4–111.0)	0.18
Diameter of FL	−100.0 (−100.0 to −100.0)	−100.0 (−100.0 to −99.8)	0.76
Area of TL	153.7 ± 69.9	36.4 (8.1–102.5)	0.23
Area of FL	−100.0 (−100.0 to −100.0)	−100.0 (−100.0 to −100.0)	0.17
**Plane 2**			
Diameter of TL	90.39 (8.6–332.3)	89.9 (25.1–225.1)	0.90
Diameter of FL	−100.0 (−100.0 to −95.8)	−100.0 (−100.0–95.34)	0.72
Area of TL	267.9 ± 79.1	170.2 ± 30.8	0.99
Area of FL	−100.0 (−100.0 to −93.5)	−100.0 (−100.0 to −93.5)	0.99
**Plane 3**			
Diameter of TL	56.8 (11.4–118.3)	32.9 (1.4–121.0)	00.35
Diameter of FL	−100.0 (−100.0 to −100.0)	−100.0 (−100.0 to −16.4)	**0.18**
Area of TL	128.7 ± 33.9	146.2 ± 49.8	0.82
Area of FL	−100.0 (−100.0 to −67.5)	−48.5 (−100.0 to −3.6)	**0.08**

*FL, false lumen; TL, true lumen. “-” stands for decrease in the diameter and area. PP analysis, per-protocol analysis. Plane 1 was the aorta plane with the maximal diameter. Plane 2 was the aorta plane where the TL was most constricted by the FL. Plane 3 was the aorta plane of the distal end of the stent*.

The two groups showed comparable clinical success rates- Ankura II: 51/52 (98.1%) vs. Ankura: 54/56 (96.4%), *p* = 1.000. The complete thrombosis rate was significantly increased at 12 months compared with that at the 1-month follow-up in both groups. However, no significant difference in the thrombosis rate was found between the two groups.

The percentage change in the diameter and area 12 months after surgery was calculated before statistical analysis. In Plane 1 and Plane 2, the TL was expanded, and the FL diameter was decreased but without significant difference (Table 4). In Plane 3, Ankura II stents showed a possible advantage in facilitating thrombosis of the FL, with a decrease trend in diameter of −100.0% (−100.0 to −100.0%) (vs.−100.0% (−100.0% to −16.4%) for the Ankura; *p* = 0.18) and a decrease trend in area of −100.0% (−100.0% to −67.5%) (vs. −48.5% (−100.0% to −3.6%) for the Ankura; *p* = 0.08). The 12-month follow-up results demonstrated that the Ankura II and Ankura groups both performed well in expanding the TL and aortic remodeling was improved in patients treated with Ankura II.

## Discussion

In the present trial, the Ankura II Thoracic Stent Graft System was safe and effective in treating TBADs. In terms of primary outcomes, no migration, collapse, fracture, or thrombosis of the stent occurred. Comparisons of the 1-month and 12-month MAEs showed no significant difference between Ankura and Ankura II. And comparison of 1-month and 12-month secondary outcomes further demonstrated that Ankura II had possible advantages in promoting FL thrombosis. Both groups had a low 1-month all-cause mortality rate (Ankura II 1.7 vs. Ankura 4.5%) and a high 12-month survival rate (Ankura II 94.2 ± 2.8 vs. Ankura 92.9 ± 3.1%) compared to other studies (7). Lower mortality rates are believed to result from multiple factors, including improvement in the stent design, good preoperative preparation and blood pressure control, and restricted inclusion criteria that excluded complicated cases.

Despite the benefits of TEVAR over conventional open repair, its adverse effects, reintervention rates and endoleaks events still raise concerns, particularly in situations with an unfavorable anatomy (9, 19). Notably results published by other centers suggest that the diameter mismatch between the stent and aortic lesions may be related to the occurrence of RTAD (10). In our trial, because of a smaller distal end diameter design compared to Ankura (27.7 ± 3.3 mm for Ankura II vs. 29.6 ± 3.1 mm for the Ankura; *p* < 0.001), the distal end of the stent in the Ankura II group complied better with the anatomy of the aorta. And follow-up results coincidentally showed that one RTAD and another dissection rupture occurred in the Ankura group. Furthermore, published results reveal that the rate of secondary intervention after elective TEVAR ranges from 0 to 32.3%, 1 month to 5 years, respectively (12, 20–22). Most were performed because of endoleaks or device-related complications. In our study, the reintervention rates for Ankura II group at 1 month is 1.7 and 7.7% after 12 months follow up.

Given the fact both endoleaks and reintervention rate are low in our study, we think it is most likely because of the improvements in the device design, thus resulting a better adherence between the graft and aorta, which also reminds surgeon to pay more attention to stent graft instructions when planning TEVAR to get a better adherence. But our follow up time is only 1 year, our data is far from sufficient to make any conclusions. the long-term reintervention rates and endoleaks are still required in following up and future studies are still warranted to prove the above conclusion.

Another interesting finding of current study is we found Ankura II stents could facilitate FL thrombosis better compared to the first-generation stent near the stent distal end area. As is all known, remodeling of the aorta after stent deployment, particularly FL thrombosis, is critical for stent stability and long-term outcomes (11). More importantly, the diameter of FL is negatively correlated with survival in patients undergoing endovascular therapy for chronic TBADs (12). Our trial showed the complete FL thrombosis rates at 12 months are 60.8 and 73.2% in the Ankura II and Ankura groups, respectively. Moreover, both groups had successful and satisfactory TL expansion rates and FL reducing rates. Ankura II stents also showed a possible advantage in facilitating FL thrombosis near the stent distal end area, with a decrease in the diameter (85.7 ± 4.4 vs. 66.9 ± 7.8%; *p* = 0.048) and a decrease in area (77.4 ± 7.9% (vs. 49.6 ± 10.1%). We speculate it is because of the tapered design of Ankura II and leading to better adhesion between the stent and aorta. And the availability of longer-length devices may also contribute to these more favorable outcomes (174.1 ± 19.2 mm for Ankura II vs. 165.6 ± 14.0 mm for the Ankura, *p* = 0.003). However, close postoperative surveillance by imaging with CTA or magnetic resonance angiography (MRA) remains extremely important for long-term results.

Several unavoidable limitations exist in this study. First, as a non-inferiority study, the non-inferiority margin (0.1) is a little wider compared to the incidence of MAEs (0.05), which makes the results underpower. Second, the follow up rate of current study is low (82.8% at 1 month) and thus have many missing data, making either ITT analysis or PP analysis have the type II or type I bias. And our total follow up time is limited (1 year), which is insufficient to make statistical conclusion. Finally, we haven't distinguished the complicated and uncomplicated TBAD during the patients recruiting process. Given the fact the complicated and uncomplicated TBAD behave differently in terms of MAE (23, 24), this is one major weakness of our study.

Overall, the 1-year outcomes from our prospective, randomized, multicenter study demonstrate that Ankura II shows comparable results to Ankura for treating TBADs and yields low mortality rates, MAEs and reintervention rates. Additionally, Ankura II shows a possible advantage in FL thrombosis. More data are warranted to evaluate the long-term effects of Ankura II and its performance in complicated cases.

## Data Availability Statement

The raw data supporting the conclusions of this article will be made available by the authors, without undue reservation.

## Ethics Statement

The studies involving human participants were reviewed and approved by Institutional Review Board of each participating institution. The patients/participants provided their written informed consent to participate in this study.

## Author Contributions

CS and HH designed the study. HH, WF, WG, ML, EX, SG, XC, ZX, SY, JH, XD, ZW, WL, QZ, and FG collected the clinical data. HH, QL, LW, ML, XL, and JW analyzed and interpreted the data. HH and CS wrote the draft. CS, HH, WF, WG, ML, EX, SG, XC, ZX, SY, JH, XD, ZW, WL, QZ, QL, LW, ML, XL, JW, and FG edited the final version. CS guided all the process. All authors contributed to the article and approved the submitted version.

## Funding

This was an industry-sponsored trial. The authors received no funding for the analysis, interpretation, or writing of the manuscript.

## Conflict of Interest

The authors declare that the research was conducted in the absence of any commercial or financial relationships that could be construed as a potential conflict of interest.

## Publisher's Note

All claims expressed in this article are solely those of the authors and do not necessarily represent those of their affiliated organizations, or those of the publisher, the editors and the reviewers. Any product that may be evaluated in this article, or claim that may be made by its manufacturer, is not guaranteed or endorsed by the publisher.
