# Supplementary material for: 1-Year Outcomes of a Multicenter Randomized Controlled Trial of the Ankura II Thoracic Endoprosthesis for the Endovascular Treatment of Stanford Type B Aortic Dissections
Source: Front Cardiovasc Med. 2022 Mar 15;9:805585. doi: 10.3389/fcvm.2022.805585 (PMC8964940; doi:10.3389/fcvm.2022.805585)
Supplement: Supplementary file 1 [file Data_Sheet_1.docx]

***Supplementary data***

Materials and methods

Device description

The deployment process is easy and specially designed to be operator friendly, as the delivery system is provided with a two-handle mechanism. The operator releases the clasping system of the stent graft in two movements: ﬁrst, by removing the safety buckle, and then by pulling back the tip releaser. This release mechanism allows the delivery system to be precisely transported, and after graft deployment and before tip capture release, it is still possible to make small ﬁnal positional adjustments because of nitinol scaffolding, which is internal to the double ePTFE membrane. The sheath has excellent resistance to bending and can be used for complex vascular access without bending. The tip and sheath core are integrally injection molded to prevent the tip from falling off and to improve convey or safety.

Results

Baseline anatomy features of aortic lesions

Among all patients, 21.9% (14/64) and 25.0% (17/68) of the patients in the Ankura II and control groups, respectively, had cases complicated with aneurysm formation (Supplementary Table 1). In the Ankura II group, 9.4% (6/64) of patients exhibited ischemia of the branch arteries. In contrast, in the control group, 11.8% (8/68) patients exhibited ischemia of the branch arteries. At the same time, data for the first tear site, the proximal landing zone, including its diameter, distance to LSA and landing zone length, were collected and compared between the two groups; other information, including the proximal TL diameter, distal TL diameter, proximal FL diameter and distal FL diameter were similarly evaluated. The above baseline anatomic etiologies were analyzed with proper statistical methods, and no significant differences were found between the two groups for any anatomic factors described above.

Surgery procedures

One hundred thirty-two patients received TEVAR, and at least one stent was implanted for each patient (Supplementary Table 2). A second stent was implanted in 4 patients in the Ankura II group and 6 patients in the control group, indicating that patients with longer arterial lesions were evenly distributed between the two groups. For the first stent that was used, the proximal diameter of the stent was 32.6±3.3 mm for Ankura II (vs 32.9±2.7 mm for the control, p=.874), the distal diameter was 27.5±3.2 mm (vs 29.4±3.0 mm for the control, p<.001), and the length of the stents was 173.4±19.0mm (vs 164.5±14.2 mm for the control, p=.003). For the second stent that was used, no significant differences were found between the two groups in terms of proximal stent diameter, distal diameter and stent length. General anesthesia was applied in 78.1% (50/64) of the patients (vs 82.4% (56/68) of the controls, p=.542), while the others received local anesthesia. The median surgery time was 67.0 min (vs 62.0 min for the controls, p=.256), the median DSA time was 15 min (vs 12.0 min for the controls, p=.618), and the median time for stent deployment was 2.0 min (vs 2.0 min for the controls, p=.780). The contrast agent used for the Ankura II group was 124.2±46.6 ml (vs 119.8±47.2 ml for the control, p=.605). The total time of the operation, the time for DSA, the time of stent release and the amount of contrast agent were not significantly different between the two groups, suggesting that the surgical difficulty of and operative skills required for the two stent grafts were comparable.
