# Supplementary material for: 1-Year Outcomes of a Multicenter Randomized Controlled Trial of the Ankura II Thoracic Endoprosthesis for the Endovascular Treatment of Stanford Type B Aortic Dissections
Source: Front Cardiovasc Med. 2022 Mar 15;9:805585. doi: 10.3389/fcvm.2022.805585 (PMC8964940; doi:10.3389/fcvm.2022.805585)
Supplement: Supplementary file 2 [file Table_1.docx]

**Supplementary Table 1 Baseline anatomical features of the aortic lesions**

|  | Ankura II % (n/N) | Control % (n/N) | P value |
| --- | --- | --- | --- |
| ***Aortic dissection*** | 100% (64/64) | 100% (68/68) | 1.000 |
| Aneurysm formation | 21.9% (14/64) | 25.0% (17/68) | .672 |
| Ischemia of branch arteries | 9.4% (6/64) | 11.8% (8/68) | .656 |
| First tear site |  |  | .264 |
| Aortic arch | 14.5% (9/62) | 19.4% (13/67) |  |
| Descending aorta | 85.5% (53/62) | 79.1% (53/67) |  |
| Abdominal aorta (supra-coeliac trunk) | 0% (0/62) | 0% (0/67) |  |
| Abdominal aorta (infra-renal) | 0% (0/62) | 0% (0/67) |  |
| Iliac artery | 0% (0/62) | 0% (0/67) |  |
| Other | 0% (0/62) | 1.5% (1/67) |  |
| Proximal landing zone |  |  |  |
| Diameter (mm) | 29.4±3.1 | 29.1±3.7 | .628 |
| Distance to LSA (mm) | 26.1±20.7 | 25.7±23.2 | .906 |
| Landing zone length (mm) | 24.6±14.6 | 23.0±12.3 | .502 |
| Proximal TL D (mm) | 23.3±7.0 | 22.8±7.8 | .679 |
| Distal TL D (mm) | 16.7±7.4 | 15.8±7.6 | .488 |
| Proximal FL D (mm) | 17.1±12.8 | 18.9±12.4 | .401 |
| Distal FL D (mm) | 13.4±10.8 | 15.3±10.7 | .310 |

LSA: left subclavian artery, D: diameter. TL: true lumen, FL: false lumen
