# Supplementary material for: 1-Year Outcomes of a Multicenter Randomized Controlled Trial of the Ankura II Thoracic Endoprosthesis for the Endovascular Treatment of Stanford Type B Aortic Dissections
Source: Front Cardiovasc Med. 2022 Mar 15;9:805585. doi: 10.3389/fcvm.2022.805585 (PMC8964940; doi:10.3389/fcvm.2022.805585)
Supplement: Supplementary file 3 [file Table_2.docx]

**Supplementary Table 2 Stents and surgical procedure information**

|  | Ankura II | Control |  |
| --- | --- | --- | --- |
| Stents |  |  | P value |
| First stent used | N=64 | N=68 |  |
| Proximal D. (mm) | 32.6±3.3 | 32.9±2.7 | .874 |
| Distal D. (mm) | 27.5±3.2 | 29.4±3.0 | **<.001** |
| Length (mm) | 173.4±19.0 | 164.5±14.2 | **.003** |
| Second stent used | N=4 | N=6 |  |
| Proximal D. (mm) | 32.8±3.5 | 32.5±2.1 | .833 |
| Distal D. (mm) | 28.1±2.8 | 28.4±2.1 | .841 |
| Length (mm) | 160.0±0.0 | 165.5±14.5 | .464 |
| Anesthesia |  |  |  |
| General | 78.1% (50/64) | 82.4% (56/68) | .542 |
| Local | 21.9% (14/64) | 17.6% (12/68) |  |
| Surgery time (min) |  |  |  |
| Median | 67.0 | 62.0 | .256 |
| Q1～Q3 | 50.0～90.0 | 50.0～90.0 |  |
| Min～Max | 30.0～290.0 | 15.0～140.0 |  |
| Time for stent deployment (min) |  |  |  |
| Median | 2.0 | 2.0 | .780 |
| Q1～Q3 | 1.0～3.0 | 1.0～3.0 |  |
| Min～Max | 0.0～20.0 | 0.2～22.0 |  |
| Contrast agent (ml) | 124.2±46.6 | 119.8±47.2 | .605 |
| DSA time (min) |  |  |  |
| Median | 15 | 12.0 | .618 |
| Q1～Q3 | 6.0～22.0 | 5.0～20.0 |  |
| Min～Max | 2.0～58.0 | 2.0～80.0 |  |

Q: Quartile, Min: minimal, Max: maximal, D: diameter. DSA: digital subtraction angiography
